# Gene characterization and molecular pathway analysis of reverse thermosensitive genic male sterility in eggplant (*Solanum melongena* L.)

**DOI:** 10.1038/s41438-019-0201-z

**Published:** 2019-11-01

**Authors:** Bing Li, Xueping Chen, Yanrong Wu, Aixia Gu, Jingjing Zhang, Shuangxia Luo, Xiurui Gao, Jianjun Zhao, Xiuqing Pan, Shuxing Shen

**Affiliations:** 1 Key Laboratory of Vegetable Germplasm Innovation and Utilization of Hebei, Collaborative Innovation Center of Vegetable Industry in Hebei, College of Horticulture, Hebei Agricultural University, Baoding, 071000 China; 2 Institute of Cash Crops, Hebei Academy of Agriculture and Forestry Sciences, Shijiazhuang, 050051 China

**Keywords:** Plant breeding, Plant reproduction, Plant development

## Abstract

The naturally occurring mutant eggplant line 05ms was identified with reverse thermosensitive genic male sterility (rTGMS), but its temperature-responsive fertility mechanisms remain largely unknown. Here, we studied the flower morphology, anther cellular structure, and genome-wide gene expression of this rTGMS line. Candidate genes for thermosensitive male sterility during the microspore development of 05ms and the temperature-insensitive line S63 under low-temperature (LT) and high-temperature (HT) conditions were identified. Under LT, tapetum cells were vacuolated and had delayed disintegration in 05ms. RNA-seq analysis indicated that DEGs were enriched in the KEGG pathways ‘plant hormone signal transduction’, ‘starch and sucrose metabolism’, and ‘phenylpropanoid biosynthesis’. We identified two genes, *4CLL1* (*Sme2.5_00368.1_g00010.1*) and *CKI1* (*Sme2.5_10056.1_g00002.1*), which could potentially regulate eggplant anther development and may be candidate genes for rTGMS. Finally, we propose a working model of anther abortion for rTGMS in eggplant. *CKI1* responds to LT stress and causes expression changes in genes related to anther development, such as *4CLL1*, and the cellular structure of the tapetum becomes abnormal, causing male sterility. The findings of this study explain the underlying molecular mechanisms of male sterility in eggplant rTGMS lines.

## Introduction

Eggplant (*Solanum melongena* L.) is an important vegetable that is cultivated throughout the world. Male sterile lines not only provide key breeding tools for hybrid seed production but also offer essential materials for studying crop reproductive development^1^. Hybrids of eggplant are usually obtained via hand emasculation and pollination. Therefore, creating male sterile resources and studying the mechanism of male sterility in eggplant are essential for improving and selecting sterile lines, as well as providing a theoretical basis and technical support for the utilization of heterosis.

In general, heritable male sterility is divided into the following three types^2^: cytoplasmic male sterile (CMS), nuclear male sterile (GMS), and cytoplasmic-nuclear male sterile (CGMS). CMS in vegetable crops, such as cabbage^3^, radish^4^, and pepper^5^, has been extensively utilized. Several CMS lines in eggplant have been reported and are usually produced by distant crossing^6^. However, studies concerning the application of male sterile lines are very limited because of several difficulties, including the narrow background of germplasm resources, the need for specific restoration genes, and the lack of restorer lines or advantages for the hybrid combinations.

Compared with CMS, the advantages of environment-sensitive genic male sterile (EGMS) lines: thermosensitive genic male sterile (TGMS) lines, photoperiod-sensitive genic male sterile (PGMS) lines, and humidity-sensitive genic male sterile (HGMS) lines, include simpler procedures for breeding and hybrid seed production^7,8^. The discovery of the first natural photoperiod-sensitive male sterile rice plant in 1973 initiated a new era of heterosis utilization^9^. Since 2012, two lines of hybrid rice have been developed and now account for almost 30% of the rice planted in China^10^. In addition, there are reverse photoperiod- and thermosensitive genic male sterile (P/TGMS) lines, which have opposite phenotypes from normal P/TGMS lines. For example, J207S is a reverse TGMS (rTGMS) line, and it is sterile in low-temperature (LT) but fertile in high-temperature (HT)^11^. TGMS in solanaceous crops such as pepper^12^ and tomato^13^ have also been reported. In 1983, the tomato thermosensitive sterility mutant *sl-2* was reported^14^ to be sterile at HT. Excessive auxin and abscisic acid at HT were cited as important causes of anther abortion. In 2013, a thermosensitive cytoplasmic male sterility (TCMS) pepper material was reported;^15^ it was sterile at HT, but fertility was restored at LT. Abnormal development of tapetal cells, along with the biosynthesis of callose and its deposition to cell walls, may be the primary cause of male sterility^16^. However, the mechanism of anther abortion of eggplant rTGMS is still unclear. In 2005, our research group found a spontaneous male sterile mutant of eggplant^17^. After several generations of breeding and selection, the stable male sterile line 05ms was obtained, which is different from all the other male sterile types in eggplant and is a rTGMS type. Exploitation of line 05ms will facilitate new avenues to utilize eggplant heterosis.

Reports on the mechanism of male sterility have been mainly focused on rice and *Arabidopsis*. Approximately 150 rice male sterility genes have been mapped (http://www.ricedata.cn/ontology/ontology.aspx?ta = TO:0000437), and 24 male sterility genes of *A. thaliana* have been described (https://www.arabidopsis.org/). Among the photoperiod- and thermosensitive genic sterility genes reported for rice, including *thermosensitive male sterility-5* (*tms5*), *photoperiod-sensitive male sterility-3*/*photoperiod-thermosensitive male sterility 12–1* [*pms3*/(*p/tms12–1*)] and *photoperiod-sensitive male sterility-1* (*pms1*), most have been used in two-line hybrid breeding, and more than 95% of two-line hybrid rice used *tms5* as a sterility gene in China^18^. The *tms5* mutation was found to cause TGMS through a loss of RNase Z^S*1*^ function^19^. In *Arabidopsis*, the double mutant *myb33*/*myb65* had hypertrophy of the tapetum and the middle layer of the cell wall in the premeiotic stage, but fertility was restored under higher light or lower temperature conditions^20^. In *Arabidopsis*, *RECEPTOR-LIKE KINASE2* (*RPK2*) mutants developed only three cell layers, the middle layer was missing, the tapetum was hypertrophied, and the endothecium had inadequate thickening and lignification^21^. TGMS studies in rice showed that *thermosensitive genic male sterile 10* (*tms10)* regulated anther development above 24 °C, and a *tms10* mutant caused anther abortion because of abnormal tapetum degradation^22^. *Carbon starved anther* (*CSA*) is mostly expressed in the tapetum and directly regulates the expression of *monosaccharide transferase 8* (*MST8*) in anthers in the *csa*-based PGMS line of rice^23^. RNA-seq studies of cotton anthers revealed that the sugar and auxin signaling pathways played important roles in anther development and resistance to HT^24^. Currently, research on the mechanisms of male sterility in eggplant is focused on aspects relating to genetics, cytology, physiology, and biochemistry; however, there are limited reports of the molecular mechanism(s) of male sterility. Notably, with the rapid development of biotechnology, sequencing of 1093 Mb of the genome was performed by Japanese scholars in 2014 and has provided a reference for the study of eggplant functional genomics, promoting the progress of eggplant research into the whole-genome era^25^. Yang et al.^26^ analyzed the flower buds of the eggplant line CMS EP26A and its maintainer line EP26 at different developmental stages by RNA-seq technology and found that differentially expressed genes (DEGs) were often enriched in redox, sugar and amino acid metabolism, transcriptional regulation and other pathways. BSA-seq sequencing was performed on *ms-3*, a male sterile mutant controlled by a single recessive nuclear gene of cucumber, and a nonsynonymous mutant SNP site was found in the gene *Csa3M006660*, which encodes a homologous domain finger protein and plays an important role in the early pollen development of cucumber^27^. Transcription factors also play an extremely important role in anther development^28^. Members of the bHLH transcription factor family regulate the anther development of plants by activating or inhibiting the spatiotemporal specific expression of a series of genes related to flower development^29^.

In this study, the eggplant rTGMS line 05ms and its wild-type fertile line S63 were used as research materials. The morphological differences in flowers were examined, and cytological observations of cell wall development in anthers were performed. Our specific objectives were the following: (1) to identify the major biological processes and metabolic pathways that regulated fertility in the eggplant rTGMS line 05ms; (2) to discover the molecular regulatory mechanism of fertility based on low and high temperatures; (3) to identify candidate genes associated with fertility transformation; and (4) to provide an account of comprehensive gene expression information at the transcriptional level. This study will provide a theoretical basis for the creation of excellent thermosensitive male sterility lines and the breeding of new varieties with stronger heterosis in eggplant.

## Results

### Morphological observations and comparison of floral organs

**Fig. 1:**
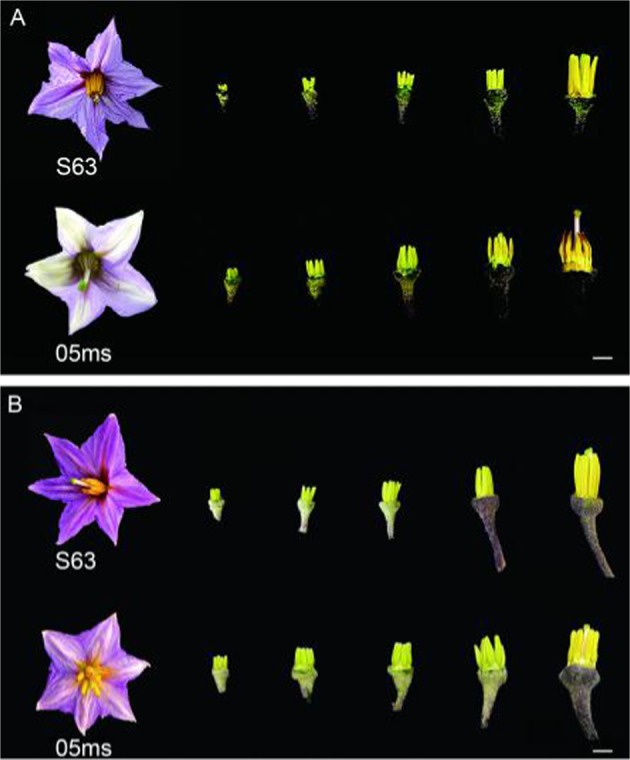
Morphological comparison of floral organs in rTGMS line 05ms and fertile line S63 of eggplant (*Solanum melongena* L.). **a** sterile period. **b** fertile period. Bar = 5 mm

There were no significant differences in the development of floral organs during the fertile period between S63 and 05ms. However, during the sterile period, the size of sterile flowers in 05ms was significantly smaller than that in S63, the petal color was lilac, and the anthers were brown and shriveled. In contrast, the petals of S63 were purple, and it had golden anthers (Fig. 1a, b).

The degree of flower opening and the mean petal and anther lengths of 05ms were significantly lower than those of S63 (Table S1). However, the diameter of the floral buds and ovaries of 05ms were both significantly greater than those of S63 flowers. The anther length of 05ms was only 53% of that of S63, and this was the primary difference between the two lines.

### Anther tissue cytology

**Fig. 2:**
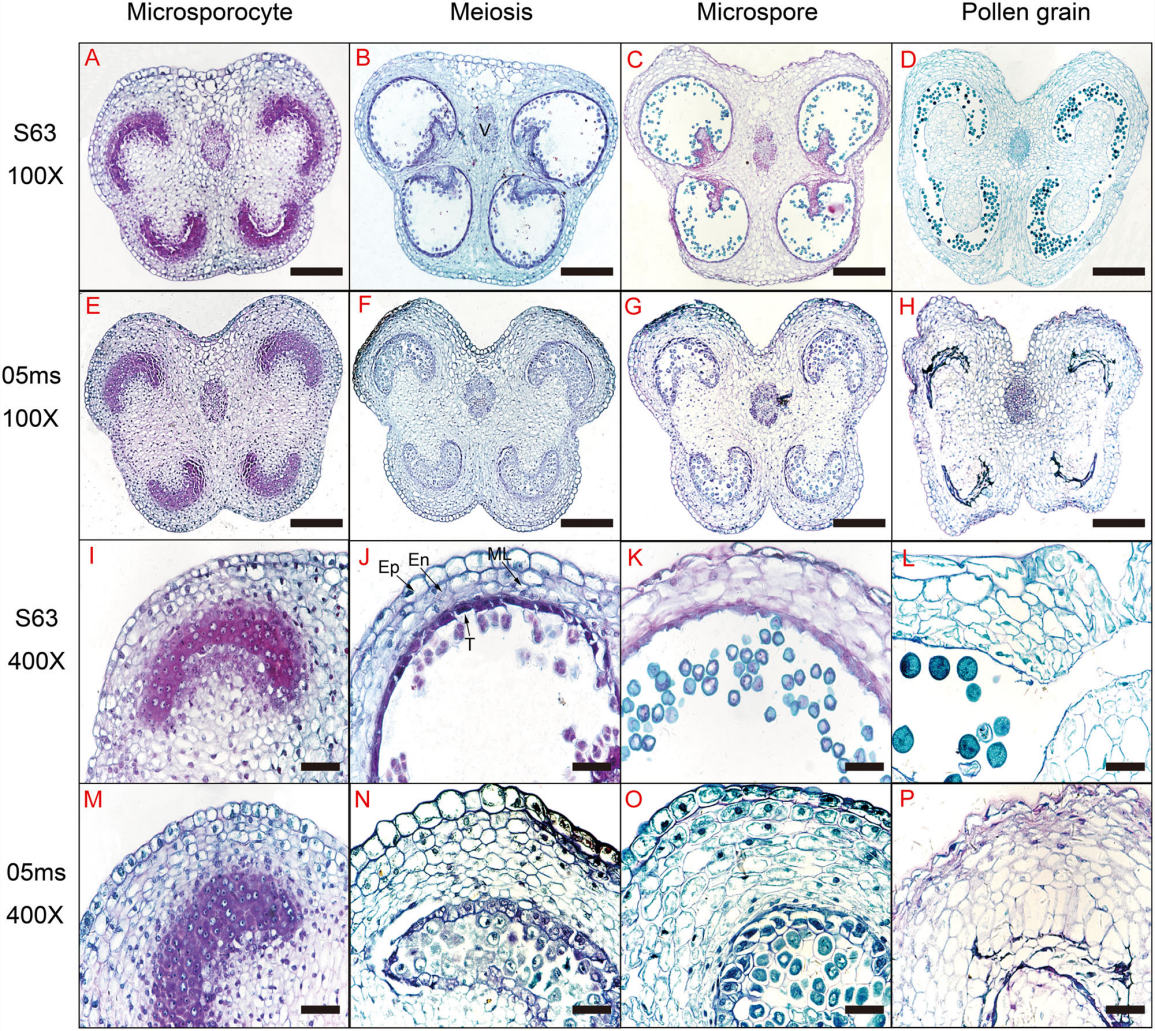
Microstructural comparison of anthers in rTGMS line 05ms and fertile line S63 of eggplant (*Solanum melongena* L.). The plants (side) and the developmental stages (top) are indicated. V vascular bundle, EP epidermis, En inner wall, ML middle layer, and T tapetum. **a**–**h** 100×, Bar = 500 μm. **i**–**p**: 400×, Bar = 100 μm

Observation of anther tissue in S63 (Fig. 2) showed that pollen mother cell (PMC) development was normal during meiosis and was followed by the formation of tetrads. During this process, the middle layer of the cells disintegrated. Tapetum cells were well developed at this time and had nonvacuolated cytoplasm and abundant inclusions (Fig. 2j). The tapetum started to degenerate in the late phase of meiosis and completely disappeared when the pollen grains were formed (Fig. 2k, l).

**Fig. 3:**
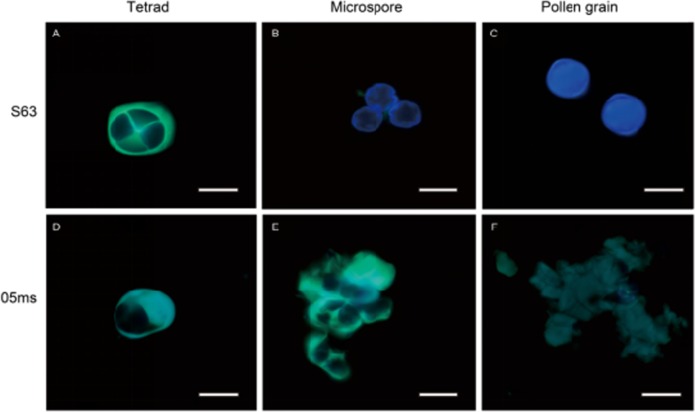
Callose comparison of anthers in rTGMS line 05ms and fertile line S63 of eggplant (*Solanum melongena* L.). **a**–**c**, S63; **d**–**f**, 05 ms; 400×; Bar = 100 μm. **a**, **d** Tetrad stage; **b**, **e** Microspore stage; **c**, **f**, Pollen grain stage

In contrast with S63, 05ms had the following five primary types of abnormalities during the development of anther tissues: (1) 2–4 additional cell layers were present with wall thickness approximately two times that of S63 (Fig. 2m–p); (2) the PMC remained in the early stage of meiosis and did not form pollen (Fig. 2m–p); (3) the tapetum cells began to vacuolate at the meiosis stage and eventually became highly vacuolated; (4) degradation of the tapetum cells was either delayed or did not occur (Fig. 2h, p); and (5) the contents of the anthers disintegrated, and either only cytoplasmic debris and the nuclei remained, or the entire anther aborted and was present only as an empty chamber (Fig. 2h, p).

Observations of callose changes during anther development by fluorescence staining indicated that callose was deposited uniformly around the tetrads, and they separated from each other during the tetrad period in S63 (Fig. 3a). In contrast, the PMCs of 05ms were surrounded by callose (Fig. 3d). Subsequently, the callose of S63 degraded, released the microspores, and the fluorescence signal diminished and finally disappeared (Fig. 3b, c). However, in 05ms, the callose accumulated, yielding a very strong fluorescence signal, and was not degraded (Fig. 3e). During the maturation of the pollen grains, the fluorescent signal of the callose in 05ms disappeared with the gradual degradation of the microspores (Fig. 3f).

### Differentially expressed gene screening

RNA-seq provided 674,245,518 clean reads (Tables S2, 3); therefore, the quality of the samples in this sequencing was sufficient for the subsequent analyses. The qRT-PCR and RNA-seq results were consistent, and R^2^ was above 0.80 (Fig. S1).

**Fig. 4:**
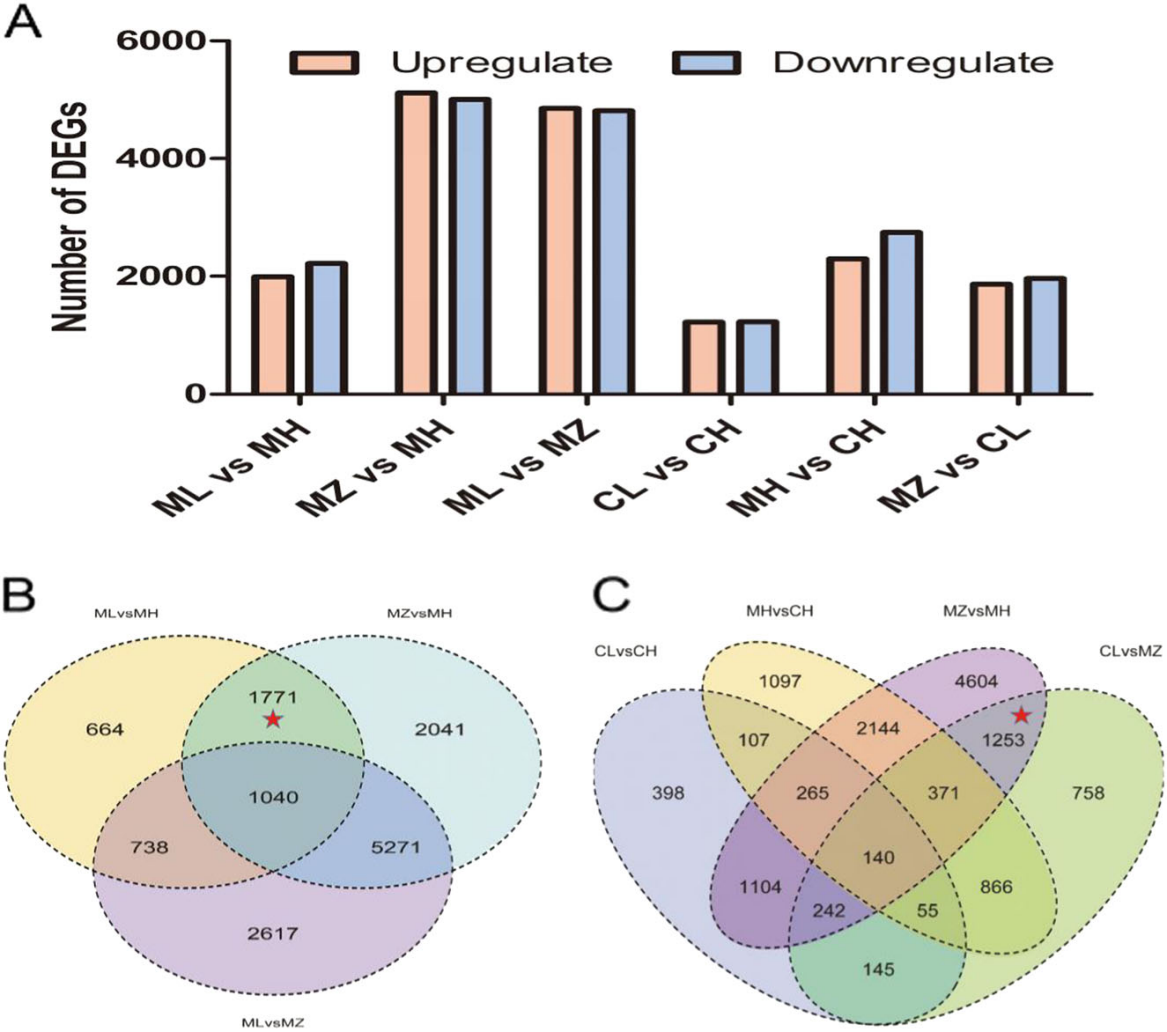
Analysis of the differentially expressed genes (DEGs) between different combinations of comparisons in the thermosensitive genic male sterile line 05ms and fertile line S63 of eggplant (*Solanum melongena* L.). **a** Numbers of DEGs in different comparisons. **b** Venn diagram showing the overlapping DEGs in 05ms. **c** Venn diagram showing the overlapping DEGs in S63 and 05ms. ML spring sterile period at LT in 05ms, MH summer fertile period at HT in 05ms; MZ autumn sterile period at LT in 05ms, CL autumn fertile period at LT in S63 CH summer fertile period at HT in S63

According to fragments per kilobase per million (FPKM), the DEGs between CL (fertile period at autumn LT) and CH (fertile period at summer HT) were the lowest (2456 DEGs) among the different growth periods of S63 (Fig. 4a). The DEGs among the three different growth stages of 05ms were significantly greater in number than those of S63, and 4213 DEGs were found between ML (the spring LT sterile period) and MH (the summer HT fertile period). However, the maximum number of DEGs was between MH and MZ (the autumn LT sterile period), and the expression of many genes changed in MZ. There were also 9666 DEGs in MLvsMZ, which might have been caused by their different growth and developmental stages.

There were 2811 common DEGs between MLvsMH and MZvsMH (Fig. 4b), which could be related to fertility and temperature. After removing 1040 genes in common with MLvsMZ, the remaining 1771 genes were associated with stable sterility under LT. We found that 1116 of the 1771 DEGs went from upregulated to downregulated, whereas 655 DEGs went from downregulated to upregulated in the growth period of ML-MH-MZ. This finding indicated that the 1771 genes changed regularly with the different temperatures, and most of the genes were downregulated during the sterile period. There were 2006 common genes between MZvsMH and CLvsMZ, which could be related to fertility. MZvsMH was different in 05ms at different temperatures (sterile period vs fertile period), and CLvsMZ was different between 05ms and S63 at the same temperature (05ms vs S63) (Fig. 4c). After removing 753 genes shared by CLvsCH or MHvsCH, the remaining 1253 genes were associated with stable male sterility under LT. We found that 569 of the 1253 genes were upregulated and 684 were downregulated in MZvsMH, whereas 571 were upregulated and 682 were downregulated in MZvsCL. Thus, 567 upregulated and 680 downregulated genes were shared by both MZvsMH and MZvsCL.

There were 240 eggplant genes with more than 60% homology to rice and 66 eggplant genes homologous to *Arabidopsis*, of which 43 genes were common (Fig. S2). Cluster analysis of the expression levels of these 43 genes revealed that 11 genes were minimally expressed during the sterile period but highly expressed during the fertile period. Among them, three genes in 05 ms were expressed over 50 X more in the HT and LT periods (Table 1) and included the following genes: *Sme2.5_00368.1_g00010.1* (*4-coumarate-CoA ligase-like 1*, *4CLL1*); a gene named *AT1G62940* (*ACOS5*) in *Arabidopsis*; and a gene named *LOC_Os04g24530* (*OsACOS12*) in rice. The other two genes were *Sme2.5_05369.1_g00001.1* and *Sme2.5_00681.1_g00009.1*, both of which belong to the *MYB35* gene family; they are named *TDF1*/*MYB35* in *Arabidopsis* and *OsTDF1* in rice. The expression levels of the above three genes were 4.59–648.21–2.38, 0.45–50.18–0.17, and 0.04–64.10–0.04, respectively, in the MZ-MH-MZ period.

**Table 1:** The expression levels of 11 common genes related to male sterility in rice, *Arabidopsis* and eggplant

Gene ID	ML_fpkm	MH_fpkm	MZ_fpkm	CL_fpkm	CH_fpkm	Blast Swiss-Prot
Sme2.5_10326.1_g00002.1	0.03	0.13	0.05	0.10	0.13	−//−
**Sme2.5_00368.1_g00010.1**	**4.59**	**648.21**	**2.38**	**50.94**	**368.55**	**4CLL1_ARATH 4-coumarate--CoA ligase-like 1**
Sme2.5_00128.1_g00007.1	0.21	0.87	0.18	0.20	0.49	GAM1_ORYSI Transcription factor GAMYB
**Sme2.5_05369.1_g00001.1**	**0.45**	**50.18**	**0.17**	**2.51**	**10.62**	**MYB35_ARATH Transcription factor MYB35**
Sme2.5_00007.1_g00018.1	0.01	0.47	0.03	0.07	0.17	MYB26_ARATH Transcription factor MYB26
Sme2.5_07055.1_g00007.1	0.00	0.63	0.02	0.03	0.03	MYB5_ARATH Transcription repressor MYB5
Sme2.5_01766.1_g00003.1	0.01	0.05	0.01	0.01	0.01	GAM1_ORYSJ Transcription factor GAMYB
Sme2.5_00994.1_g00003.1	8.44	15.82	8.41	11.45	12.99	MYB4_ORYSJ Myb-related protein Myb4
**Sme2.5_00681.1_g00009.1**	**0.04**	**64.10**	**0.04**	**8.71**	**26.99**	**MYB35_ARATH Transcription factor MYB35**
Sme2.5_00096.1_g00009.1	0.00	0.04	0.00	0.00	0.01	RAX3_ARATH Transcription factor RAX3
Sme2.5_00705.1_g00006.1	0.00	0.02	0.00	0.00	0.02	ODO1_PETHY Protein ODORANT1

The bold values were indicated important key genes

The expression levels of 11 genes related to tapetum development changed significantly with temperature (Table 2). Among them, seven genes were contained in 1771 or 1253 genes. The expression levels of *TAPETAL DEVELOPMENT and FUNCTION1* (*TDF1*), *ETERNAL TAPETUM1* (*EAT1*) and *ABORTED MICROSPORE* (*AMS*) were significantly lower at LT than at HT, and the change range of 05ms was significantly higher than that of S63. However, the expression level of *AMS* was not significantly different between the two lines at HT, although the difference was almost 35 times at LT.

**Table 2:** Gene expression levels during tapetum development

Gene	Gene ID	ML_fpkm	MH_fpkm	MZ_fpkm	CL_fpkm	CH_fpkm
DYT1	Sme2.5_05438.1_g00004.1	51.58	16.08	8.58	69.00	104.96
TDF1/MYB35	Sme2.5_00681.1_g00009.1	0.04	64.10	0.04	8.71	26.99
TIP2/EAT1	Sme2.5_00114.1_g00007.1	7.28	45.2	5.64	14.78	76.27
**AMS**	**Novel01177**	**1.41**	**214.97**	**0.56**	**35.90**	**216.97**
TDR	Sme2.5_03795.1_g00002.1	3.07	6.21	2.62	2.43	3.28
RPK2	Sme2.5_01106.1_g00005.1	0.03	3.18	0.02	0.02	0.48
GAMYB	Sme2.5_00346.1_g00023.1	0.43	2.08	0.23	0.20	0.92
MYB65	Sme2.5_04770.1_g00001.1	8.54	8.33	10.39	8.75	6.19
MYB33	Sme2.5_02281.1_g00003.1	23.57	25.15	34.33	35.18	24.24
EMS1/EXS	Sme2.5_13301.1_g00003.1	5.31	4.24	7.09	12.82	11.25
TPD1	Sme2.5_02440.1_g00005.1	14.89	5.38	8.15	5.29	9.05

The bold values were indicated important key genes

### Functional analysis of differentially expressed genes

Gene Ontology (GO) analysis was used to analyze the major biological functions of the 1771 genes in 05ms during the different temperature periods, and 1253 DEGs in S63 and 05ms were identified (Fig. S3). Although the DEG numbers were different according to Blast2GO, similar functions and proportions were found between the 1771 and 1253 DEGs. The biological process category accounted for 48% of the DEG functions, and among them, 23% were primarily enriched in cellular processes (GO: 0009987) and 22% in metabolic processes (GO: 0008152). In the cell composition category, 19% of the DEGs were enriched in cell components (GO: 0005623), and 19% were enriched in the cell (GO: 0005623) term. In the molecular function category, 46% of the DEGs were enriched in binding (GO: 0005488), and 38% were enriched in catalytic activities (GO: 0003824).

**Fig. 5:**
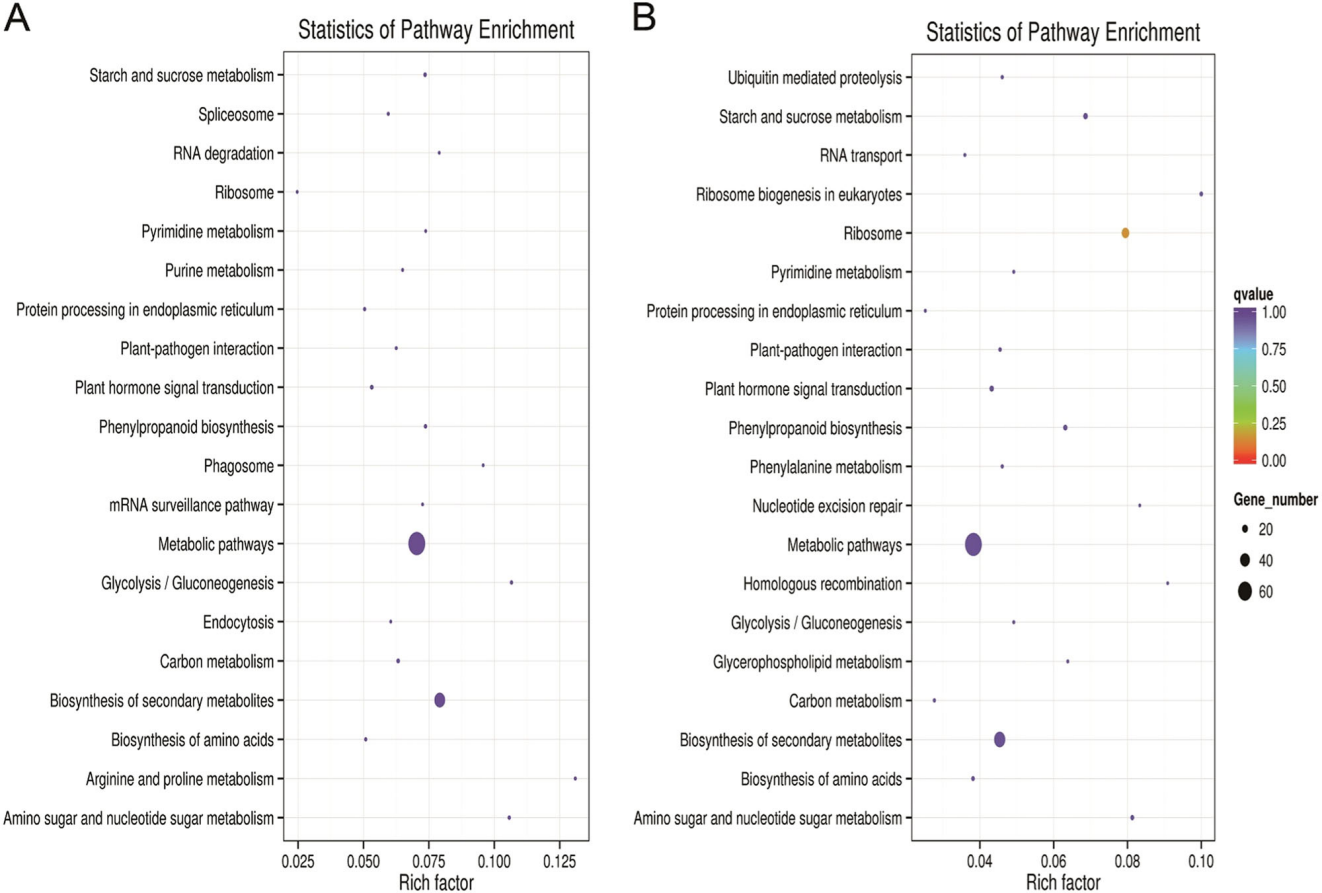
Enriched KEGG pathway scatter plots for DEGs in thermosensitive genic male sterile line 05ms (**a** 1771 DEGs) and fertile line S63 (**b**, 1253 DEGs) of eggplant (*Solanum melongena* L.)

To analyze the biological pathways, 1771 and 1253 DEGs were annotated to reference pathways in the Kyoto Encyclopedia of Genes and Genomes (KEGG) (Fig. 5). The 1771 DEGs were assigned to 108 KEGG pathways, and the top 20 are shown in Table S4. Many DEGs that were enriched in these pathways were related to ‘metabolic pathways’, ‘biosynthesis of secondary metabolites’, ‘carbon metabolism’, ‘plant hormone signal transduction’, and ‘starch and sucrose metabolism’. Specifically, the ‘metabolic pathways’ DEGs included those for ‘carbon metabolism’ (16), ‘starch and sucrose metabolism’ (15), and ‘amino sugar and nucleotide sugar metabolism’ (13). The pathway for ‘biosynthesis of secondary metabolites’ included ‘phenylpropanoid biosynthesis’ (14), ‘purine metabolism’ (10), and ‘pyrimidine metabolism’ (9).

This group of 1253 DEGs was enriched in 95 KEGG pathways. Among the top 20 pathways (Table S5), the most highly represented groups were related to ‘metabolic pathways’, ‘biosynthesis of secondary metabolites’, ‘ribosomes’, ‘plant hormone signal transduction’, and ‘phenylpropanoid biosynthesis’. The ‘metabolic pathways’ encompassed members of DEGs for ‘starch and sucrose metabolism’ (14), ‘amino sugar and nucleotide sugar metabolism’ (10), and ‘carbon metabolism’ (7). The ‘biosynthesis pathway of secondary metabolites’ involved members of the ‘phenylpropanoid biosynthesis’ (12), ‘phenylalanine metabolism’ (7), and ‘pyrimidine metabolism’ (6).

Comparison of 1771 and 1253 DEG enrichment pathways was performed, and the common pathways included ‘metabolic pathways’, ‘biosynthesis of secondary metabolites’, ‘plant hormone signal transduction’, ‘starch and sucrose metabolism’, ‘carbon metabolism’, and ‘phenylpropanoid biosynthesis’. The pathways were all involved in energy and nutrient supply and distribution and thus affected the cell and cell wall development. The ‘ribosome pathway’ was enriched in 1253 DEGs but not in 1771 DEGs and was related to adversity stress. Among the 1771 and 1253 DEGs, 215 genes were common to both groups, and 2809 DEGs were annotated in KEGG pathways. All 2809 DEGs were found in MH and MZ, and 1926 of them were expressed differently between ML and MH. The upregulated and downregulated pathways in the sterile stages were analyzed (Fig. S4).

### Transcription factors

**Fig. 6:**
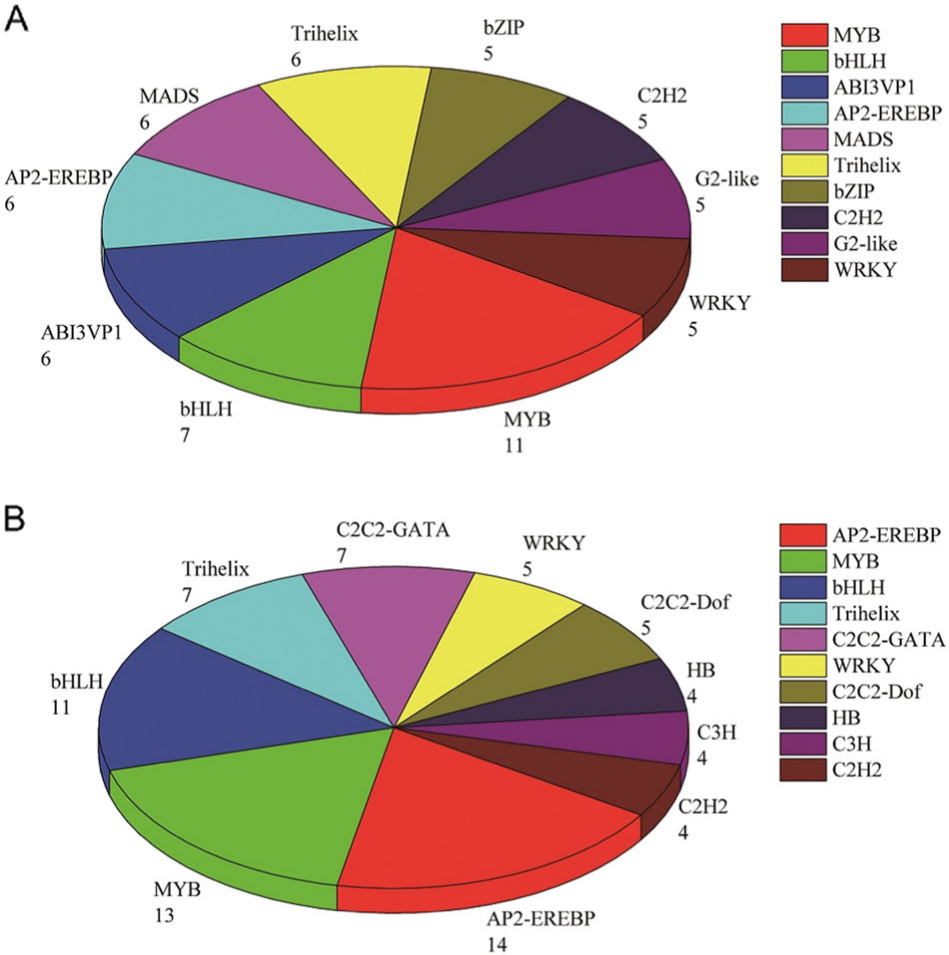
Transcription factors identified in the groups of 1771 DEGs and 1253 DEGs. **a** TF among the 1771 DEGs; **b** TFs among the 1253 DEGs

There were 114 transcription factors (TFs) among the 1771 DEGs (Fig. 6-a) and 127 TFs in 1253 DEGs (Fig. 6-b). The most represented TF families were the same between the 1771 and 1253 DEGs and included *MYB*, *bHLH*, *AP2/EREBP*, and *Trihelix*. These TFs regulated the expression of DEGs by promoting or inhibiting their expression.

### BSA-seq analysis

After sequencing, a total of 77.924 Gb clean bases were generated, with a coverage of 98.70% (Table S6). In total, 120,037 SNPs and 39,891 InDels were identified between the fertility and sterility pools. Finally, 16 SNPs and 10 InDels in candidate genes were found (Table 3). Among them, there were six nonsynonymous mutations, 19 upstream mutations, and one intron mutation.

**Table 3:** All 26 candidate SNP and InDel sites

Gene ID	Variant	Chrom	Pos	Ref	S63	05 ms	Alt
Sme2.5_01069.1_g00012.1	Nonsynonymous	Sme2.5_01069.1	90833	A	A	G	G
Sme2.5_01382.1_g00005.1	Nonsynonymous	Sme2.5_01382.1	51348	A	G	A	G
Sme2.5_03095.1_g00005.1	Nonsynonymous	Sme2.5_03095.1	56545	A	G	A	G
Sme2.5_03460.1_g00004.1	Nonsynonymous	Sme2.5_03460.1	25055	C	C	T	T
Sme2.5_04634.1_g00002.1	Nonsynonymous	Sme2.5_04634.1	35824	G	A	G	A
Sme2.5_10000.1_g00003.1	Nonsynonymous	Sme2.5_10000.1	17151	C	T	C	T
Sme2.5_00018.1_g00016.1	Upstream	Sme2.5_00018.1	149462	A	A	G	G
Sme2.5_00250.1_g00007.1	Upstream	Sme2.5_00250.1	29371	A	A	G	G
Sme2.5_00481.1_g00008.1	Upstream	Sme2.5_00481.1	82101	C	A	C	A
Sme2.5_01232.1_g00001.1	Upstream	Sme2.5_01232.1	1945	T	C	T	C
Sme2.5_03681.1_g00006.1	Upstream	Sme2.5_03681.1	30627	T	T	C	C
Sme2.5_04837.1_g00004.1	Upstream	Sme2.5_04837.1	27375	C	C	A	A
Sme2.5_05759.1_g00003.1	Upstream	Sme2.5_05759.1	37206	T	C	T	C
Sme2.5_06718.1_g00002.1	Upstream	Sme2.5_06718.1	26954	C	T	C	T
Sme2.5_11788.1_g00001.1	Upstream	Sme2.5_11788.1	5140	C	A	C	A
Sme2.5_00183.1_g00012.1	Upstream	Sme2.5_00183.1	105933	AT	AT	A	A
Sme2.5_00339.1_g00009.1	Upstream	Sme2.5_00339.1	93173	T	TTTCAATAG	T	TTTCAATAG
Sme2.5_00339.1_g00009.1	Upstream	Sme2.5_00339.1	93189	A	–	A	–
Sme2.5_01232.1_g00001.1	Upstream	Sme2.5_01232.1	1903	AA	–	AA	–
Sme2.5_01232.1_g00003.1	Upstream	Sme2.5_01232.1	11791	C	CGTT	C	CGTT
Sme2.5_01232.1_g00004.1	Upstream	Sme2.5_01232.1	11791	C	CGTT	C	CGTT
Sme2.5_01314.1_g00006.1	Intron	Sme2.5_01314.1	38687	A	A	–	–
Sme2.5_01656.1_g00015.1	Upstream	Sme2.5_01656.1	115606	G	G	–	–
Sme2.5_04572.1_g00005.1	Upstream	Sme2.5_04572.1	38291	–	–	A	A
Sme2.5_09964.1_g00003.1	Upstream	Sme2.5_09964.1	10606	T	–	T	–
Sme2.5_10056.1_g00002.1	Upstream	Sme2.5_10056.1	20631	ACA	–	ACA	–

‘Ref’ refers to the base of the reference genome at this position, ‘Alt’ refers to the base mutation compared to the reference genome at this position

**Fig. 7:**
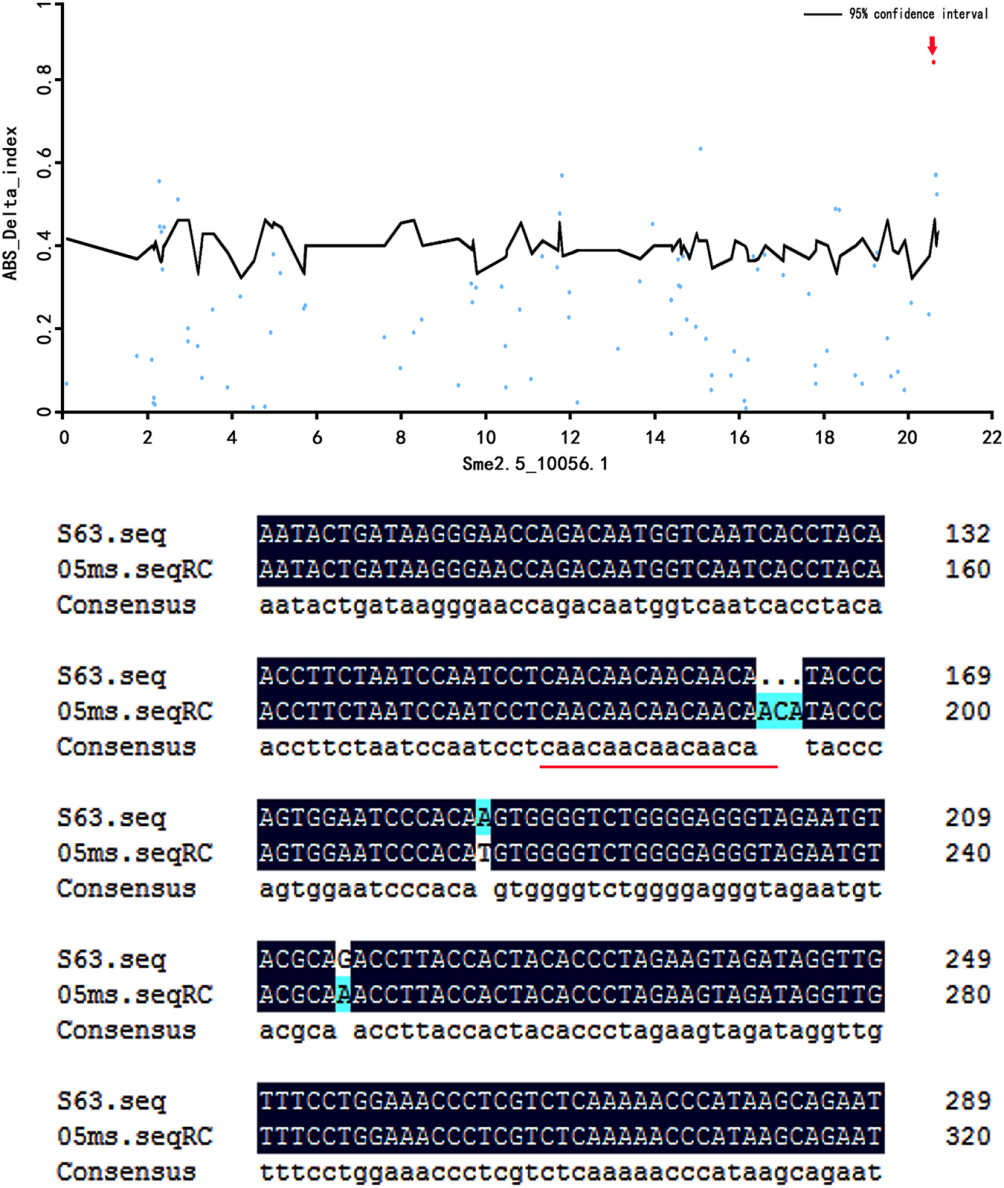
Gene *Sme2.5_10056.1_g00002.1* Manhattan plot and structure

**Fig. 8:**
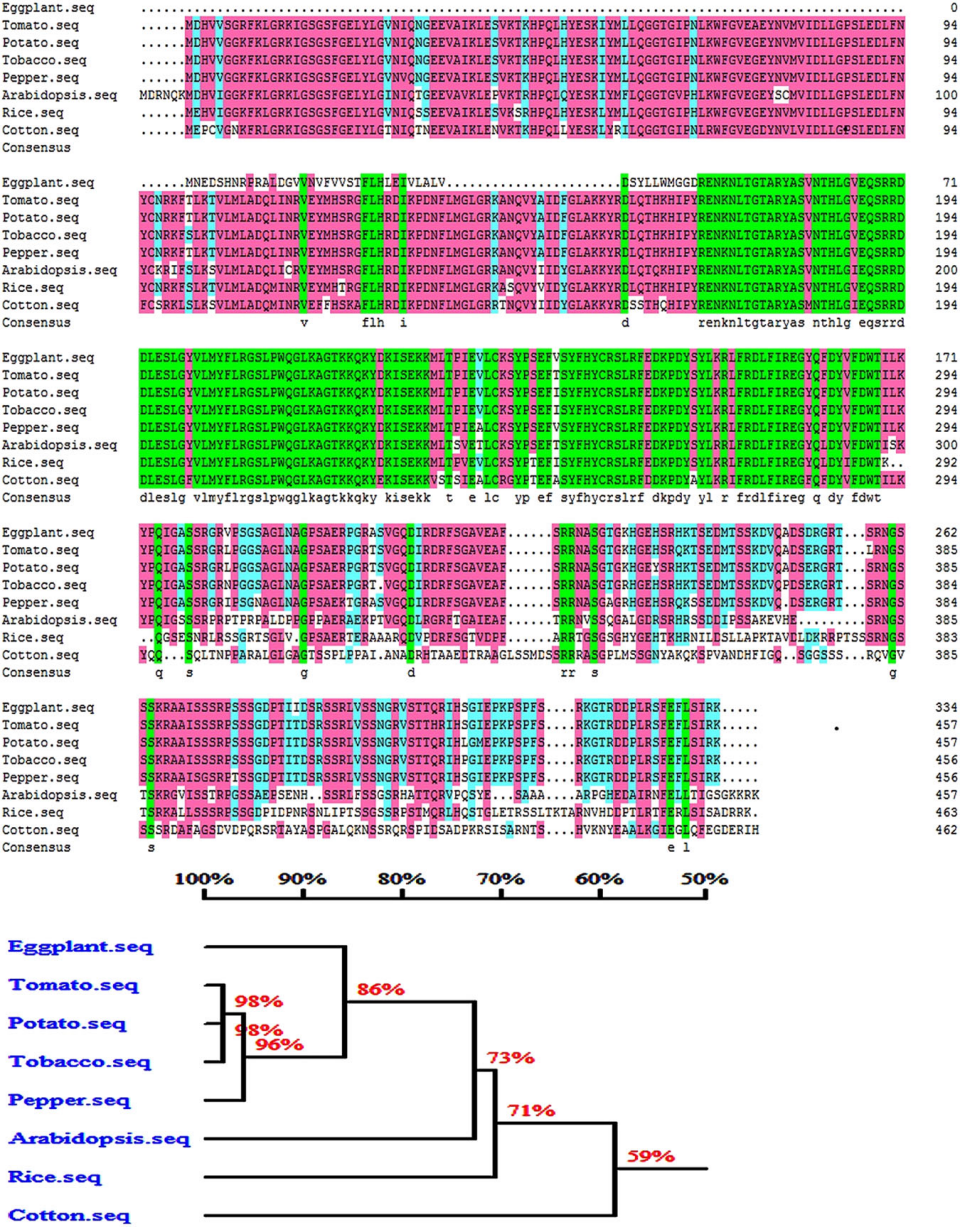
Protein homology alignment of *Sme2.5_10056.1_g00002.1* in tomato, potato, tobacco, pepper, *Arabidopsis*, rice and cotton

The 26 candidate genes screened by BSA-seq and RNA-seq were combined for analysis, and the transcript expression levels of 10 genes showed a regular change trend with the temperature changes in different growth stages (Table 4). Based on the annotation of results and protein homology comparison, a candidate gene, *sme2.5_10056.1_g00002.1*, *Casein Kinase I*, was selected. This gene may be related to thermosensitive sterility, was 5250 bp in length (Fig. 7), and was located at 15158–20407 of sme2.5_10056.1. The gene contained 10 exons and nine introns. The mutation site was located 222 bp upstream of *sme2.5_10056.1_g00002.1*, and the bases ACA was inserted into the 05ms sequence compared to S63. In this study, homologous alignments of *sme2.5_10056.1_g00002.1* were performed, and it was found to be strictly conserved (Fig. 8).

**Table 4:** Expression levels of 10 candidate genes in transcriptome sequencing

Gene ID	ML	MH	MZ	CL	CH	Swiss-Prot annotation
Sme2.5_00018.1_g00016.1	12.56	16.89	8.53	12.96	18.51	Triphosphate tunnel metalloenzyme 3
Sme2.5_04572.1_g00005.1	55.49	75.84	45.03	56.42	84.20	Protein GPR107
Sme2.5_05759.1_g00003.1	82.17	69.72	82.11	83.66	116.63	Uncharacterized protein
Sme2.5_00339.1_g00009.1	11.76	9.83	15.89	12.73	9.44	NA
**Sme2.5_10056.1_g00002.1**	**4.44**	**3.00**	**4.56**	**30.36**	**30.91**	**Casein kinase I**
Sme2.5_01314.1_g00006.1	3.18	1.36	9.08	6.56	2.90	Small heat shock protein C2
Sme2.5_01232.1_g00003.1	0.08	0.05	1.35	0.93	0.37	Protein ROOT HAIR DEFECTIVE 3
Sme2.5_01382.1_g00005.1	0.21	0.86	0.42	0.34	0.48	Uncharacterized mitochondrial protein
Sme2.5_00481.1_g00008.1	3.11	4.39	4.03	4.35	7.28	Putative formamidase
Sme2.5_03681.1_g00006.1	194.75	198.33	229.10	251.57	248.40	Ubiquitin-conjugating enzyme E2-17

The bold values were indicated important key genes

## Discussion

Male sterile mutants are excellent materials for studying the regulation of fertility gene expression and plant microevolution^30^. The rTGMS line 05ms was a spontaneous mutation in long eggplant, favorable for the production of two-line hybrids. To globally identify genes and pathways participating in eggplant anther development under LT, we performed RNA-seq analysis and obtained complete transcriptome information that allowed us to understand the relationship between anther development and LT. Our transcriptomic results offer initial data for functional studies of anther development in this species. We studied the transcriptional regulation mechanisms of eggplant floral buds at the time of meiosis under different temperatures in 05ms and S63. Combining these data with KEGG pathway analyses, we found that DEGs were mainly enriched in ‘plant hormone signal transduction’, ‘starch and sucrose metabolism’, and ‘phenylpropane biosynthesis’ pathways. We identified two genes, *4CLL1* (*Sme2.5_00368.1_g00010.1*) and *CKI1* (*Sme2.5_10056.1_g00002.1*), which could potentially regulate eggplant anther development.

The tapetum is highly sensitive to environmental stimulation^31^. A mutant of *AMS* displays abnormally enlarged tapetum cells and aborted microspore development^29^. Genome-wide coexpression analysis revealed that *AMS* can directly regulate 23 genes implicated in the degradation of callose from pollen tetrads, formation of phenolic compounds, and sporopollenin synthesis^29^. Interestingly, the protein sequences of the two genes, *TDR INTERACTING PROTEIN2* (*TIP2*) and *EAT1*, were highly homologous to the gene *Sme2.5_00114.1_g00007.1. TIP2* was upstream of *TDR* and *EAT1* and was responsible for cell differentiation, and *EAT1* was accountable for the tapetum plant cell death (PCD) process^32^. Therefore, we concluded that they simultaneously controlled the development of both cell differentiation and tapetum degradation.

**Fig. 9:**
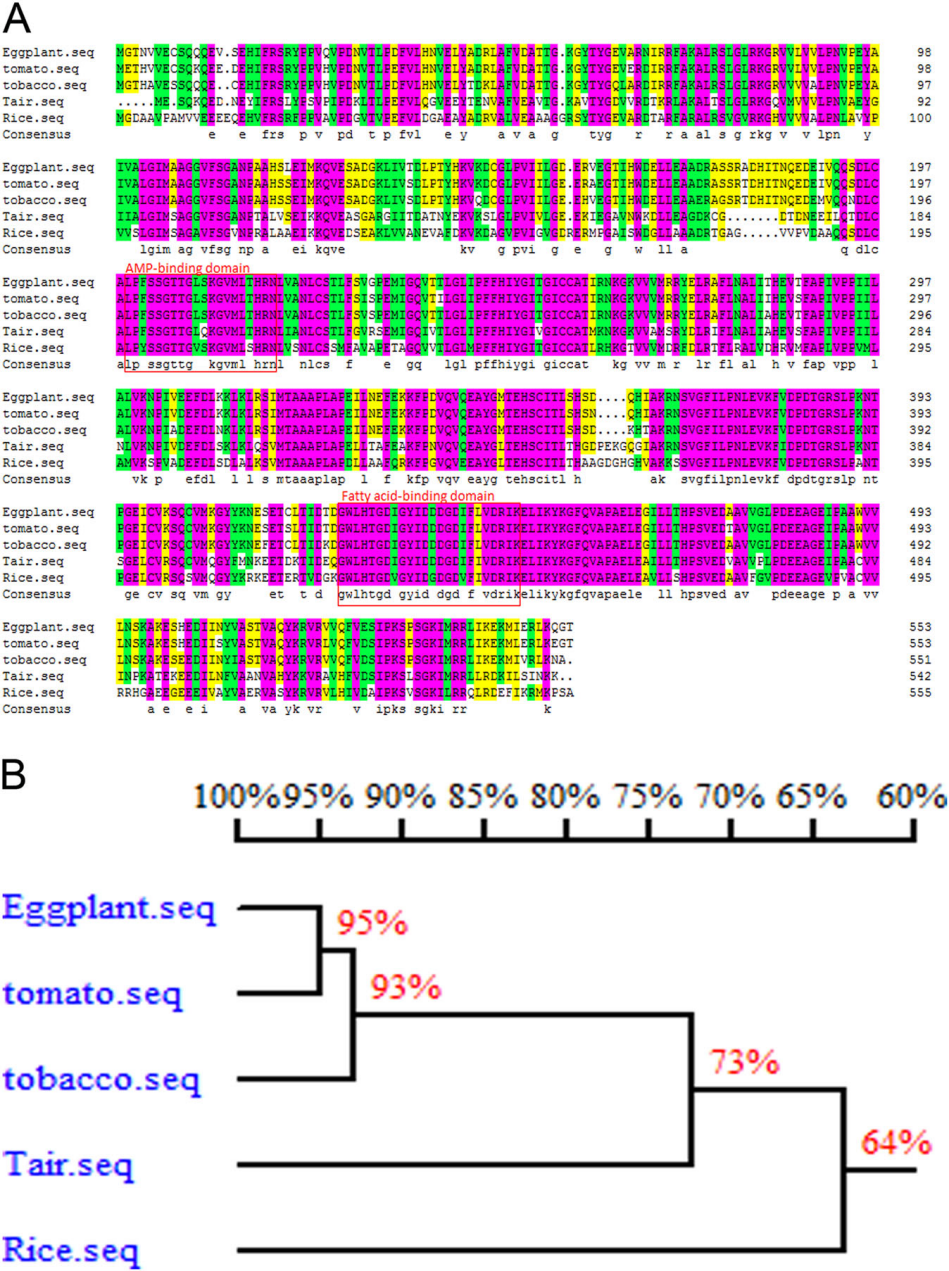
The homology alignment analysis of *4CLL1* in eggplant, tomato, tobacco, Arabidopsis (Tair) and rice. **a** BLASTP analysis; **b** Phylogenetic tree of *4CLL1* in different species

4-Coumarate: CoA ligase (4CL) is a key enzyme of phenylpropanoid metabolism in plants and is involved in lignin biosynthesis^33^. Homology alignment analysis of *4CLL1* in eggplant, tomato, tobacco, Arabidopsis, and rice revealed that the gene was highly conserved among the different species (Fig. 9). The rice *Osacos12* male sterile mutant anthers were white, shorter and smaller than those from fertile males. *OsACOS12* was expressed in tapetal cells and microspores, and the protein accumulated in tapetal cells and anther locules^34^. *OsACOS12* plays an important role in pollen wall formation and anther development in rice. The OsACOS12 enzyme catalyzed the conversion of C18:1 fatty acids to C18:1 CoA and affected tapetal PCD and male sterility, which is important for the PCD-induced degradation of tapetum and normal development of pollen in rice^35^. Cytological observations on the rTGMS line 05ms in eggplant revealed that the anthers were shrunken and necrotic, and mature pollen grains were absent. These phenomena were very similar to the rice *Osacos12* mutant^34^. Arabidopsis ACOS5, an ortholog of OsACOS12 in rice, is crucial for fertility^36^. Similar to *acos5*, the *osaocs12* mutant had no mature pollen. In tobacco, NtACOS1, an ortholog of ACOS5, was prominently expressed in the tapetum. RNAi silencing leads to male sterility^37^. The expression level of *4CLL1* in 05ms in the LT male sterile period was only 0.4–0.7% of that in the HT fertile period. *Sme2.5_00368.1_g00010.1* (*4CLL1*) may participate in the tapetal PCD of anthers and male sterility in the eggplant rTGMS line 05ms.

Therefore, LT strongly inhibited the expression of tapetum development genes, such as *TDF1*, *EAT1*, and *AMS*, in 05ms and blocked the expression of the downstream gene *4CLL1*. Additionally, a secondary metabolic disorder led to abnormal development of the middle cell layer and tapetum of the anther cell wall in eggplant rTGMS 05ms, which caused male sterility.

We speculate that *sme2.5_10056.1_g00002.1* (*CKI1*) is another candidate gene for thermosensitive sterility in eggplant. *Casein kinase I* (*CKI*) is a serine/threonine-specific protein kinase that is highly conserved in plants and animals^38^. Glucose regulates many important developmental and metabolic processes in plants, specifically ABA biosynthesis and signaling pathways^39^. In contrast, *CKI* regulates glucose perception and transport in plants^39^. Studies of *A. thaliana* showed that *atckl-2* and *atckl-7* were expressed in anthers during early development and may be the key regulatory factors for tapetal development under HT conditions^40^. In rice, a lack of *OsCKI1* affected the expression of many genes involved in signal transduction and the metabolic pathways of hormones controlling the temperature response^41^. In cotton, *GhCKI* gene expression was induced by cotton fiber temperature and caused premature abortion in tapetal cells^42^. *GhCKI* may regulate a series of genes in different pathways by inhibiting tapetal degeneration and anther wall secondary thickening, which leads to anther abortion and promotes the regulation of crop temperature tolerance^42^. In our study, the expression level of *CKI1* in the anthers of the eggplant thermosensitive sterility line 05ms was upregulated by LT stimulation. Changes in temperature caused stress responses in the plants that led to changes in the gene expression of downstream plant hormone signal transduction pathways, thus delaying programmed tapetal death and causing male sterility.

**Fig. 10:**
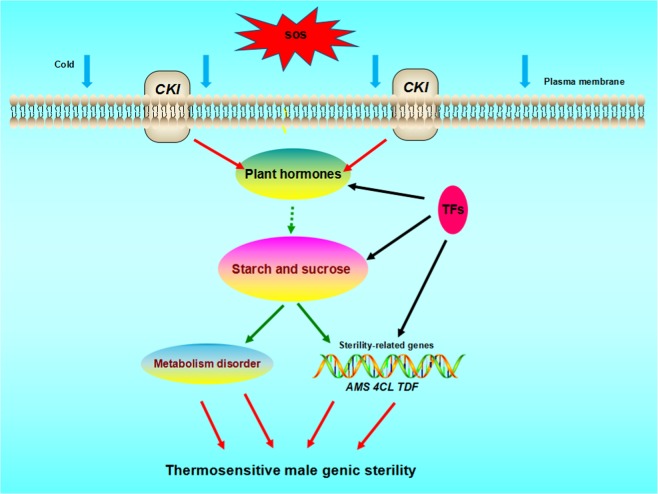
Working model of thermosensitive male genic sterility

In conclusion, we propose a possible pollen abortion model/mechanism for thermosensitive male sterility in eggplants (Fig. 10). When the eggplant thermosensitive sterile line 05ms is grown at LT, protein kinase *CKI1* expression will be upregulated. This will cause gene expression changes in the plant hormone signal transduction, sugar and starch metabolism pathways, which will decrease *4CLL1* expression. This cascade of events will culminate in abnormal tapetum development and abortion of anthers. However, transcription of the *4CLL1* gene will activate when temperatures increase, and the *4CLL1* gene expression level will sharply increase. Because of the increased expression of *4CLL1*, the tapetum develops normally, and the fertility of 05ms is restored. Our results will help to clarify the molecular mechanism of fertility transformation in eggplant TGMS and have an important impact on two-line crossbreeding of eggplant.

## Materials and methods

### Plant material and treatments

The thermosensitive male sterile line 05ms and the male-fertile line S63 of long eggplant were used as materials and were produced by the Institute of Cash Crops, Hebei Academy of Agriculture and Forestry Sciences. The original sterile 05ms plant was selected from a natural mutation of inbreeding line S63 with male sterility at LT but fertility at HT. The line 05ms was considered rTGMS, in which a daily minimum temperature of 18 °C was the primary environmental factor regulating fertility conversion^17^. On January 3, 2016, seedlings of 05ms and S63 were sown in trays in a solar greenhouse in Shijiazhuang City (114.26E, 38.03 N), Hebei province, China. At the five-leaf stage, seedlings were transplanted to a plastic tunnel. Eighty plants of each line were planted in rows with 45 cm between individuals and 80 cm between adjacent rows (Table 5). Floral buds were collected at the anther meiosis stage of development at ML (sterile period at spring LT), MH (fertile period at summer HT), and MZ (sterile period at autumn LT) for 05ms and CL (fertile period at autumn LT) and CH (fertile period at summer HT) for S63. The buds were used for transcriptomic analyses.

**Table 5:** Growth conditions and fertility characteristics of rTGMS line 05ms in eggplant (*Solanum melongena* L.)

Code	Description	Fertility	LT (°C)	HT (°C)	Date (month)
ML	Sterile stage at spring LT	Sterile	15–18	<35	4–6
MH	Fertile stage at summer HT	Fertile	>18	<35	7–9
MZ	Sterile stage at autumn LT	Sterile	10–18	<35	10–11

### Morphological observation

To compare the differences in flower morphologies between the lines 05ms and S63, at least 50 flowers were collected on the same day in April (sterile period) and September (fertile period). The characteristics of flower opening degree, petal and anther length, and diameter of floral buds and ovaries were measured with Vernier calipers (Fig. S5).

### Cytological observation

Floral buds of sterile and fertile lines were collected at the following four developmental stages in April (sterile period under LT): pollen mother cell, meiosis, microspore, and mature pollen grains (Table S7). After measuring ovary diameters and removing the sepals, the remaining floral parts were fixed in Carnoy’s fluid (absolute ethanol: glacial acetic acid = 3: 1, v/v) for callose observation and FAA fixative (70% ethanol: glacial acetic acid: formalin = 16: 1: 1, v/v/v) for paraffin sectioning. The corpus callosum was stained with 0.1% (0.1 g aniline blue/100 ml H_2_O, pH 7.0) aniline blue solution. Paraffin sections (6–8 μm) were made^43^, stained with iron vitriol-solid green, and mounted under coverslips sealed with neutral gum. Paraffin sections and callose were observed using a Ni-U fluorescence microscope (Nikon, Japan), and CCD images were acquired using a DS-Ri2 Microscope Camera (Nikon, Japan). The fluorescence of the aniline blue-stained callose was stimulated by UV (UV-2a), excitation 330–380, DM400, BA420 and photographed with an exposure time of 70 ms.

### RNA-seq analysis

Floral buds were collected from the different growth stages. After removing the sepals, the remaining floral parts were immediately flash frozen in liquid nitrogen and stored at −80 °C. Three biological replicates were collected. Total RNA was extracted using the DP441 Kit (Tiangen, China). RNA quality was assessed on 1% agarose gels, and RNA purity was determined using a Nano Photometer spectrophotometer (IMPLEN, USA). RNA concentration was measured using a Qubit RNA Assay Kit in Qubit 2.0 Fluorometer (Life Technologies, USA). RNA integrity was assessed using the RNA Nano 6000 Assay Kit of the Bioanalyzer 2100 system (Agilent Technologies, USA). RNA-seq was performed by Novogene Bioinformatics Technology Co. Ltd. (Beijing, China) on an Illumina HiSeq 4000 platform.

Raw reads were first filtered to obtain clean reads, which were aligned to the eggplant reference genome^25^ using TopHat2. HTSeq (v0.6.1) was used to count the read numbers mapped to each gene. The FPKM of each gene was calculated. Differential expression analysis (three biological replicates per condition) was performed using the DESeq R package (v1.18.0). Genes with an adjusted *P*-value <0.05, as found by DESeq, were considered differentially expressed. GO enrichment analysis of DEGs was implemented using the GOseq R package. KOBAS software was used to test the statistical enrichment of DEGs in KEGG pathways.

### Real-time quantitative RT-PCR

The total RNA used for transcriptome sequencing was also used for quantitative real-time polymerase chain reaction (qRT-PCR) analyses. Reverse transcription to produce cDNA was accomplished using the PrimeScript™ RT Reagent Kit with gDNA Eraser (code no. RR047B; TaKaRa, Japan). The first strand cDNA was reverse-transcribed from 1 µg of total RNA and diluted 1:10 with RNase-free water. Gene-specific primers were designed with Primer Premier 5.0 software (Primer, Canada). Primers are listed in Table S8, and GAPDH (GenBank: JX448342.1) was used as a reference. Each assay was performed on the ABI 7500 Real-Time PCR system (Applied Biosystems, USA) using the following thermocycling conditions: 95 °C for 30 s, followed by 40 cycles at 95 °C for 5 s and 60 °C for 40 s. All qRT-PCR analyses were performed with three replicates of a biological sample.

### BSA-seq analysis

Twenty extreme sterile and fertile plants were selected from the F_2_ segregating offspring. DNA from the leaves was extracted with the plant genomic DNA extraction kit DE-06112 (FOREGENE, China). The sterile gene pool and fertile gene pool were formed by equal mixing of samples. The constructed library was sequenced by Illumina HiSeqTM PE150 with a parent sequencing depth of 10X and a daughter pool sequencing depth of 20×/pool.

High-quality clean reads were obtained from raw reads through a series of quality control procedures. BWA (Burrows-Wheeler Aligner)^44^ was used to align the clean reads of each sample against the eggplant reference genome (ftp://ftp.kazusa.or.jp/pub/eggplant/SME_r2.5.1.fa.gz). The results were compared by SAMtools, and the subsequent bioinformatics analysis was performed^45^. GATK3.3^46^ software module was used to detect the SNP and InDel variations. ANNOVAR software was used to annotate SNPs and InDels^47^. We selected the sterile parent 05ms as the reference parent to analyze and calculate the SNP index (the frequency of SNP)^48^. We used a window size of 1 Mb and a step size of 1 Kb for the mean value of the SNP index to reflect the distribution of the SNP index among the offspring. We performed 1000 substitution tests and selected a 95% (blue) confidence level as the screening threshold. At the 95% confidence level, the window greater than the threshold was selected as the candidate interval. For the candidate polymorphic marker sites, the annotation results of ANNOVAR were extracted, and the genes that caused stop/loss, stop/gain, nonsynonymous mutation or variable splicing were selected as the candidate genes. The frequency difference analysis of offspring InDels was the same as that of the corresponding SNPs. The same analysis was performed after combining the SNP and InDel sites.

### Data analysis

The data represent the results of multiple independent experiments, and means ± SE are shown. Fluorescence quantitative experimental qPCR data were analyzed by the 2^−ΔΔct^ method^49^. All data were averaged over three replicates. Duncan’s multiple range test was conducted to compare the mean differences. Statistically significant differences were determined at *P* < 0.05 and/or *P* < 0.01.

## Supplementary information

HR-Supporting information
