# Supplementary material for: Gene characterization and molecular pathway analysis of reverse thermosensitive genic male sterility in eggplant (Solanum melongena L.)
Source: Hortic Res. 2019 Nov 1;6:118. doi: 10.1038/s41438-019-0201-z (PMC6823389; doi:10.1038/s41438-019-0201-z)
Supplement: Supplementary file 1 — HR-Supporting information [file 41438_2019_201_MOESM1_ESM.doc]

**Supporting information**

**Table S1 Comparison of flower morphology between rTGMS line 05ms and fertile line S63 of eggplant (*Solanum melongena* L.).** Values ± SD; The different lowercase letters indicate are significantly differences at P<0.05, and the different capitals are significantly different at P<0.01.

| **Material** | **Opening degree** | **Petal length** | **Anther length** | **Bud transverse diameter** | **Ovary transverse diameter** |
| --- | --- | --- | --- | --- | --- |
| S63 | 5.15±0.17 a A | 2.54±0.14 a A | 1.03±0.07 a A | 7.63±0.36 b B | 5.23±0.29 b B |
| 05ms | 4.43±0.32 b B | 2.05±0.20 b B | 0.55±0.07 b B | 9.01±0.85 a A | 6.40±0.62 a A |

**Table S2 Quality of reads for 15 RNA-Seq samples.**

| **Sample name** | **Raw**  **reads** | **Clean reads** | **Clean bases** | **Error rate**  **(%)** | **Q20**  **(%)** | **Q30**  **(%)** | **GC Content**  **(%)** |
| --- | --- | --- | --- | --- | --- | --- | --- |
| ML1 | 45,750,644 | 43,806,454 | 6.57G | 0.02 | 97.21 | 92.74 | 42.12 |
| ML2 | 51,261,330 | 47,555,150 | 7.13G | 0.02 | 95.92 | 89.43 | 42.12 |
| ML3 | 48,440,056 | 44,950,844 | 6.74G | 0.02 | 95.92 | 89.46 | 42.17 |
| MH1 | 49,008,536 | 45,044,828 | 6.76G | 0.03 | 95.49 | 88.54 | 42.25 |
| MH2 | 46,245,288 | 45,379,722 | 6.81G | 0.01 | 97.44 | 93.37 | 42.02 |
| MH3 | 49,374,898 | 42,143,908 | 6.32G | 0.03 | 95.94 | 89.31 | 41.79 |
| MZ1 | 48,994,008 | 48,045,376 | 7.21G | 0.01 | 97.64 | 93.78 | 42.23 |
| MZ2 | 48,489,646 | 44,713,256 | 6.71G | 0.03 | 95.60 | 88.84 | 42.49 |
| MZ3 | 47,984,402 | 44,247,780 | 6.64G | 0.03 | 95.61 | 88.75 | 42.43 |
| CL1 | 48,960,282 | 43,684,960 | 6.55G | 0.03 | 95.69 | 88.9 | 42.35 |
| CL2 | 49,358,154 | 48,468,434 | 7.27G | 0.02 | 97.45 | 93.41 | 42.51 |
| CL3 | 50,379,282 | 46,323,768 | 6.95G | 0.03 | 95.47 | 88.54 | 42.79 |
| CH1 | 50,633,056 | 43,439,452 | 6.52G | 0.03 | 95.93 | 89.25 | 42.45 |
| CH2 | 48,666,642 | 42,155,970 | 6.32G | 0.03 | 96.11 | 89.64 | 42.47 |
| CH3 | 45,955,650 | 44,285,616 | 6.64G | 0.01 | 97.91 | 94.20 | 42.40 |

**Table S3 Mapping statistics for 15 RNA-Seq samples.**

| **Sample name** | **Total reads** | **Total mapped** | **Multiple mapped** | **Uniquely mapped** | **Exons (%)** |
| --- | --- | --- | --- | --- | --- |
| ML1 | 43,806,454 | 36,831,295 (84.08%) | 1,073,236  (2.45%) | 35,758,059 (81.63%) | 84.90 |
| ML2 | 47,555,150 | 38,757,384 (81.5%) | 1,167,780  (2.46%) | 37,589,604 (79.04%) | 84.40 |
| ML3 | 44,950,844 | 36,568,933 (81.35%) | 1,123,774  (2.5%) | 35,445,159 (78.85%) | 84.90 |
| MH1 | 45,044,828 | 36,371,510 (80.75%) | 1,025,637  (2.28%) | 35,345,873 (78.47%) | 86.00 |
| MH2 | 45,379,722 | 38,244,873 (84.28%) | 1,133,350  (2.5%) | 37,111,523 (81.78%) | 85.20 |
| MH3 | 42,143,908 | 34,505,730 (81.88%) | 968,763  (2.3%) | 33,536,967 (79.58%) | 84.10 |
| MZ1 | 48,045,376 | 40,917,812 (85.16%) | 1,214,574  (2.53%) | 39,703,238 (82.64%) | 85.10 |
| MZ2 | 44,713,256 | 36,195,654 (80.95%) | 1,043,070  (2.33%) | 35,152,584 (78.62%) | 85.50 |
| MZ3 | 44,247,780 | 35,617,512 (80.5%) | 994,746  (2.25%) | 34,622,766 (78.25%) | 85.20 |
| CL1 | 43,684,960 | 35,408,267 (81.05%) | 1,092,584  (2.5%) | 34,315,683 (78.55%) | 84.90 |
| CL2 | 48,468,434 | 41,090,940 (84.78%) | 1,175,807  (2.43%) | 39,915,133 (82.35%) | 85.70 |
| CL3 | 46,323,768 | 37,216,680 (80.34%) | 1,036,993  (2.24%) | 36,179,687  (78.1%) | 85.00 |
| CH1 | 43,439,452 | 35,715,270 (82.22%) | 1,062,462  (2.45%) | 346,52,808 (79.77%) | 85.70 |
| CH2 | 42,155,970 | 34,745,259 (82.42%) | 1,133,570  (2.69%) | 33,611,689 (79.73%) | 85.80 |
| CH3 | 44,285,616 | 37,909,449 (85.6%) | 1,149,149  (2.59%) | 36,760,300 (83.01%) | 86.00 |

**Table S4 The top 20 KEGG pathways for the 1771 genes.**

| **Pathway_term** | **ID** | **Input number** | **Background number** | **Rich_factor** | **P-Value** |
| --- | --- | --- | --- | --- | --- |
| Metabolic pathways | sly01100 | 138 | 1961 | 0.070372259 | 0.105237849 |
| Biosynthesis of secondary metabolites | sly01110 | 82 | 1036 | 0.079150579 | 0.026757394 |
| Carbon metabolism | sly01200 | 16 | 253 | 0.063241107 | 0.541637645 |
| Plant hormone signal transduction | sly04075 | 16 | 301 | 0.053156146 | 0.790379169 |
| Starch and sucrose metabolism | sly00500 | 15 | 204 | 0.073529412 | 0.326844306 |
| Phenylpropanoid biosynthesis | sly00940 | 14 | 190 | 0.073684211 | 0.332243761 |
| Glycolysis / Gluconeogenesis | sly00010 | 13 | 122 | 0.106557377 | 0.059190373 |
| Amino sugar and nucleotide sugar metabolism | sly00520 | 13 | 123 | 0.105691057 | 0.062059453 |
| Biosynthesis of amino acids | sly01230 | 12 | 236 | 0.050847458 | 0.811812904 |
| Protein processing in endoplasmic reticulum | sly04141 | 12 | 238 | 0.050420168 | 0.820054984 |
| Arginine and proline metabolism | sly00330 | 11 | 84 | 0.130952381 | 0.025538707 |
| Plant-pathogen interaction | sly04626 | 11 | 176 | 0.0625 | 0.563259249 |
| Spliceosome | sly03040 | 11 | 185 | 0.059459459 | 0.626507879 |
| Purine metabolism | sly00230 | 10 | 154 | 0.064935065 | 0.517923381 |
| Phagosome | sly04145 | 9 | 94 | 0.095744681 | 0.160557879 |
| RNA degradation | sly03018 | 9 | 114 | 0.078947368 | 0.31204189 |
| Pyrimidine metabolism | sly00240 | 9 | 122 | 0.073770492 | 0.38009904 |
| mRNA surveillance pathway | sly03015 | 9 | 124 | 0.072580645 | 0.397342253 |
| Endocytosis | sly04144 | 9 | 149 | 0.060402685 | 0.605143138 |
| Ribosome | sly03010 | 9 | 365 | 0.024657534 | 0.999702419 |

**Table S5 The top 20 KEGG pathways for the 1253 genes.**

| **Pathway_term** | **ID** | **Input number** | **Background number** | **Rich_factor** | **P-Value** |
| --- | --- | --- | --- | --- | --- |
| Metabolic pathways | sly01100 | 75 | 1961 | 0.038245793 | 0.865920168 |
| Biosynthesis of secondary metabolites | sly01110 | 47 | 1036 | 0.045366795 | 0.360069165 |
| Ribosome | sly03010 | 29 | 365 | 0.079452055 | 0.001646755 |
| Starch and sucrose metabolism | sly00500 | 14 | 204 | 0.068627451 | 0.06456782 |
| Plant hormone signal transduction | sly04075 | 13 | 301 | 0.043189369 | 0.525779992 |
| Phenylpropanoid biosynthesis | sly00940 | 12 | 190 | 0.063157895 | 0.12655975 |
| Amino sugar and nucleotide sugar metabolism | sly00520 | 10 | 123 | 0.081300813 | 0.046551464 |
| Ribosome biogenesis in eukaryotes | sly03008 | 9 | 90 | 0.1 | 0.020079407 |
| Biosynthesis of amino acids | sly01230 | 9 | 236 | 0.038135593 | 0.679070728 |
| Plant-pathogen interaction | sly04626 | 8 | 176 | 0.045454545 | 0.48363882 |
| Ubiquitin mediated proteolysis | sly04120 | 7 | 152 | 0.046052632 | 0.478311159 |
| Phenylalanine metabolism | sly00360 | 7 | 152 | 0.046052632 | 0.478311159 |
| Carbon metabolism | sly01200 | 7 | 253 | 0.027667984 | 0.913222654 |
| Glycerophospholipid metabolism | sly00564 | 6 | 94 | 0.063829787 | 0.226682841 |
| Pyrimidine metabolism | sly00240 | 6 | 122 | 0.049180328 | 0.428208469 |
| Glycolysis / Gluconeogenesis | sly00010 | 6 | 122 | 0.049180328 | 0.428208469 |
| RNA transport | sly03013 | 6 | 167 | 0.035928144 | 0.7181145 |
| Protein processing in endoplasmic reticulum | sly04141 | 6 | 238 | 0.025210084 | 0.938791673 |
| Homologous recombination | sly03440 | 5 | 55 | 0.090909091 | 0.098636189 |
| Nucleotide excision repair | sly03420 | 5 | 60 | 0.083333333 | 0.127059594 |

**Table S6 Quality of reads for BSA-Seq samples**

| Sample | Raw bases(bp) | Clean bases(bp) | Effective rate(%) | Error rate(%) | Q20(%) | Q30(%) | GC content(%) |
| --- | --- | --- | --- | --- | --- | --- | --- |
| S63 | 12855934800 | 12839333100 | 99.87 | 0.04 | 97.6 | 93.03 | 36.81 |
| S-pool | 27473466600 | 27446630400 | 99.9 | 0.04 | 97.47 | 92.53 | 37.12 |
| F-pool | 24778950900 | 24743149200 | 99.86 | 0.04 | 97.67 | 93.01 | 37.1 |
| 05ms | 12915386100 | 12894488100 | 99.84 | 0.04 | 97.43 | 96.13 | 36.61 |

**Table S7 The length of floral bud transverse diameter at different stages of anther development in rTGMS line 05ms and fertile line S63 of eggplant (*Solanum melongena* L.).** The unit is mm. The anther developmental stages are as follows: I pollen mother cell (PMC) stage; II microspore mother cell (MMC) meiosis stage; III microspore stage; and IV mature pollen grain stage.

| **Material** | **I Pollen mother cell** | **II Meiosis** | **III Microspore** | **IV Pollen grains** |
| --- | --- | --- | --- | --- |
| 05ms | 4-5 | 5-6.5 | 6.5-7.5 | >7.5 |
| S63 | 3-4 | 4-5 | 5-6 | >6.5 |

**Table S8 Primers for qRT-PCR used to validate the RNA-Seq results.**

| **Gene ID** | **Product Length(bp)** | **Sense Primer (5' to 3')** | **Antisense Primer (5' to 3')** |
| --- | --- | --- | --- |
| Sme2.5_08308.1_g00004.1 | 151 | TGAGCGAGGACGCAAGATT | GAGTTCACCACCAGCAGCAT |
| Sme2.5_00487.1_g00003.1 | 120 | TCGCACAGAATTTGAGCAGA | GCAACTTGATCCGCAATACAT |
| Sme2.5_05775.1_g00002.1 | 119 | GAATGGGACGATAGTGGTGG | TCTGCGCTAAGGATTGAAGG |
| Sme2.5_03271.1_g00001.1 | 196 | GCTTTGCCTCAATCAACTCAT | GGGCACCATCCATGCTAAC |
| Sme2.5_05719.1_g00012.1 | 171 | ATGTATGTCAAAGTTAGCGTGGAT | ATAAGCAGGGGCAAAGTCAG |
| Sme2.5_16184.1_g00002.1 | 196 | TGCTGTTTATGTTGGGGAGA | GCCTCAGAACCTACTCAAACG |
| Sme2.5_16535.1_g00001.1 | 143 | TGTCTTGTTAAGTGCCTGTG | CTGATATTCAATAGATGTCCC |
| Sme2.5_01417.1_g00004.1 | 263 | AAATGGTCCAAGCCACTCAA | CCAGCCTCTACATATTCCCT |
| GAPDH | 260 | TCTTCACTGACAAGGACAAGGCTGC | AGTTTTCTGGGTGGCAGTCATAGCG |


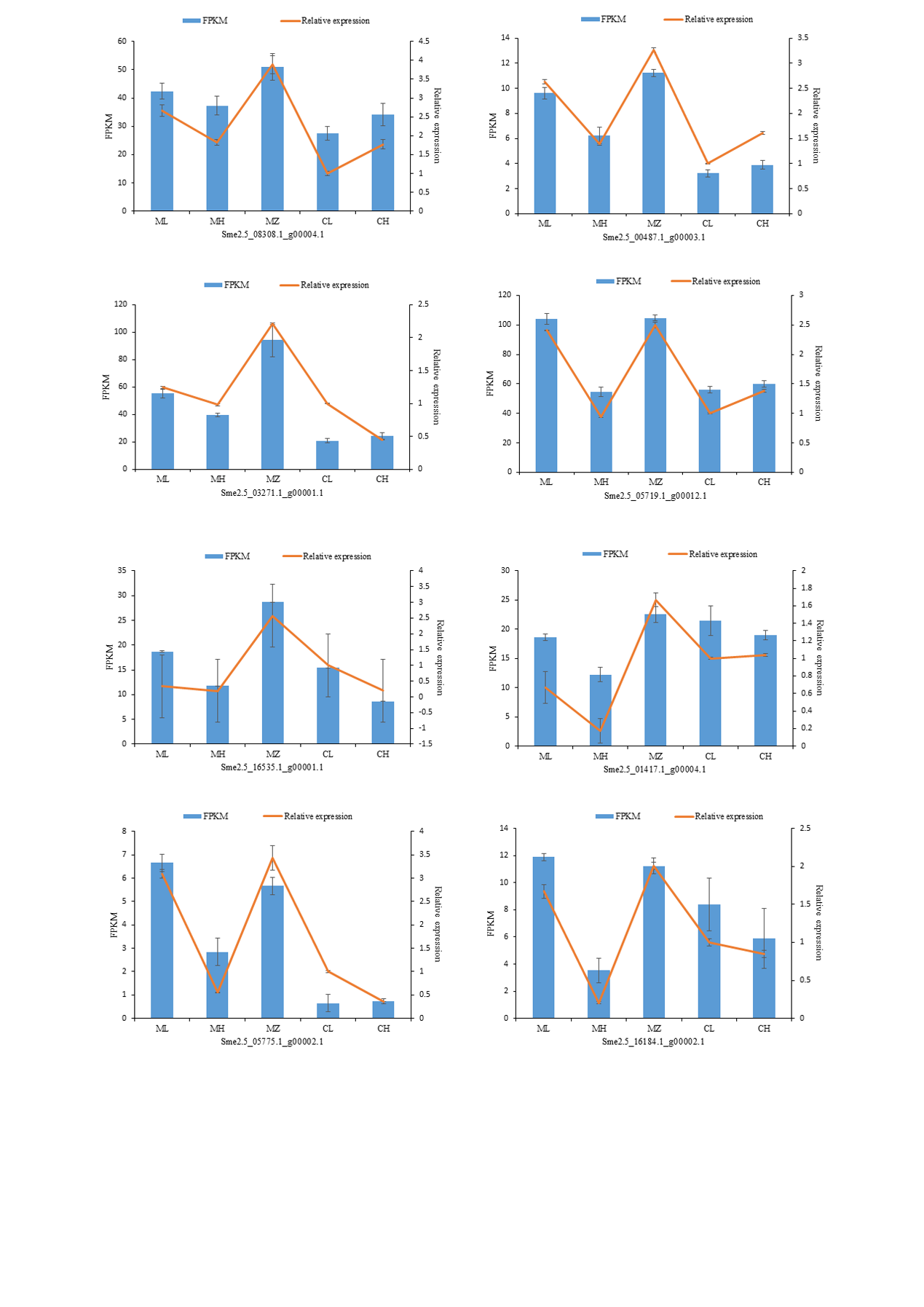
**Fig. S1 Comparison of results obtained via RNA-Seq and qRT-PCR for 8 genes expressed at different periods.**


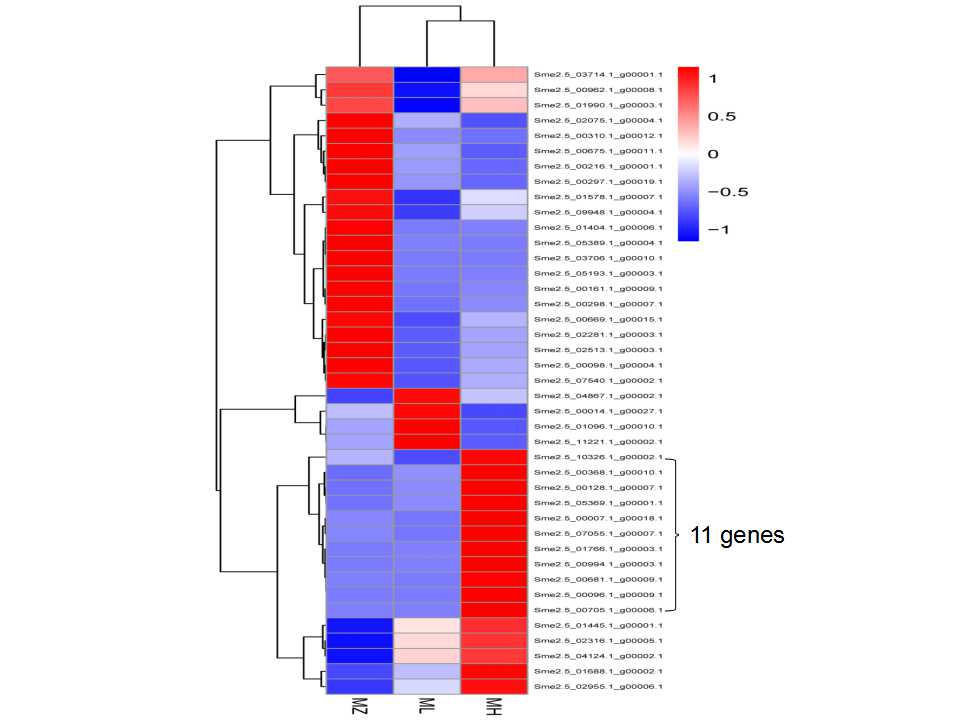


**Fig.S2 Heatmap of 43 common genes related to male sterility in rice, *Arabidopisis* and eggplant.**


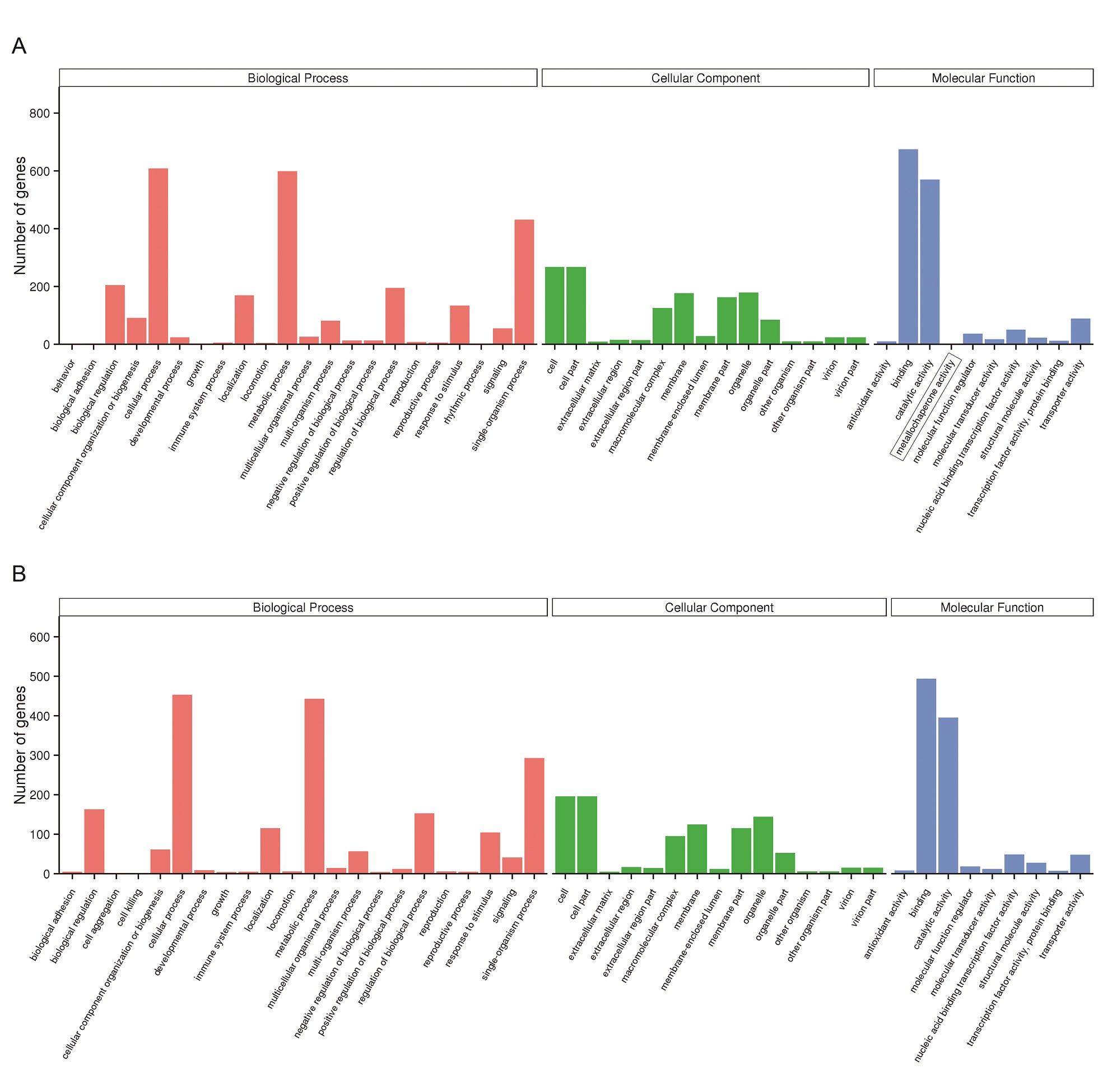


**Fig. S3 Bud differentially expressed genes by GO functional classification.**

**(A) GO classification of 1771 DEGs; (B) GO classification of 1253 DEGs.**

**
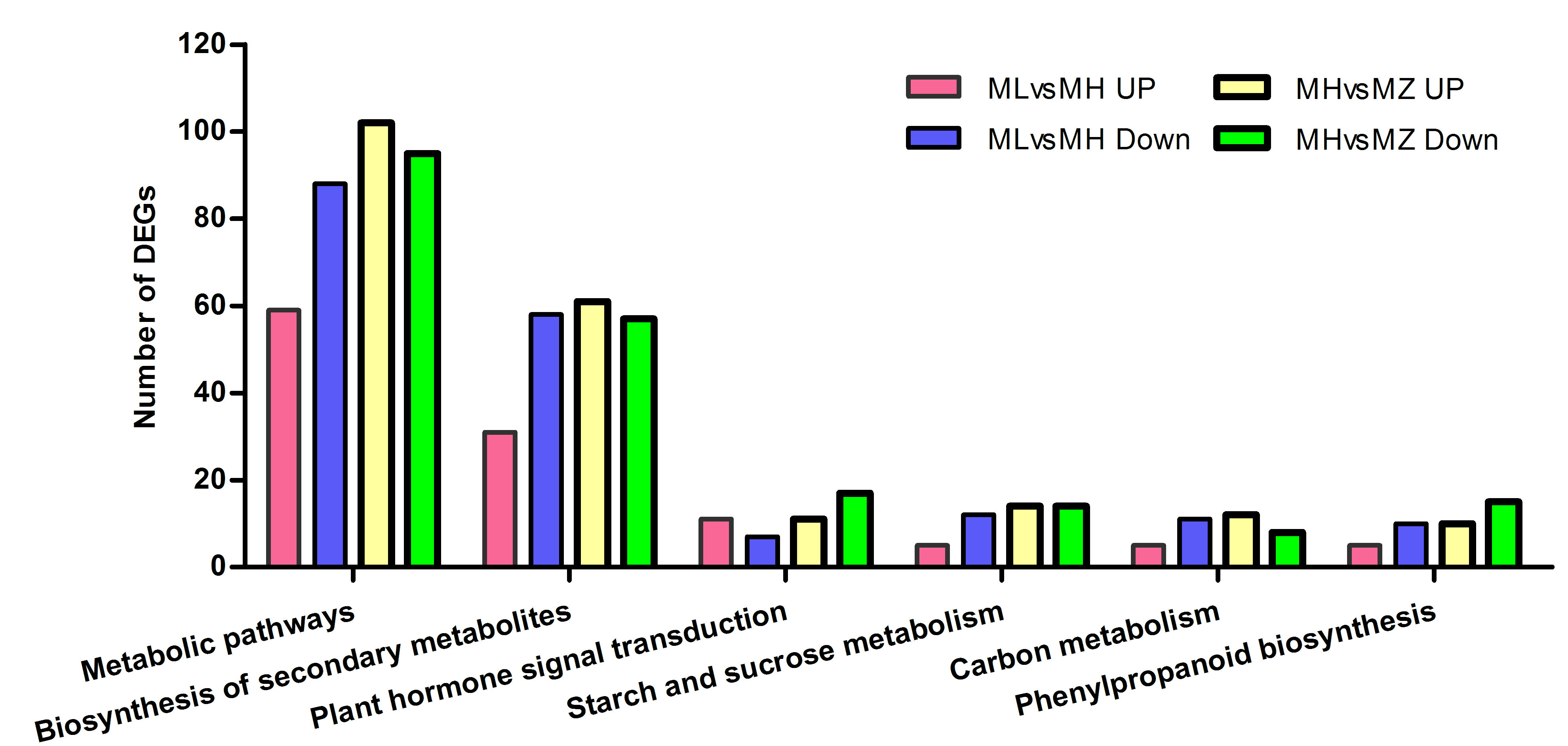
**

**Fig. S4 The number of DEGs in the most highly represented pathways of ML vs MH and MH vs MZ in 2809 DEGs.**

stage.


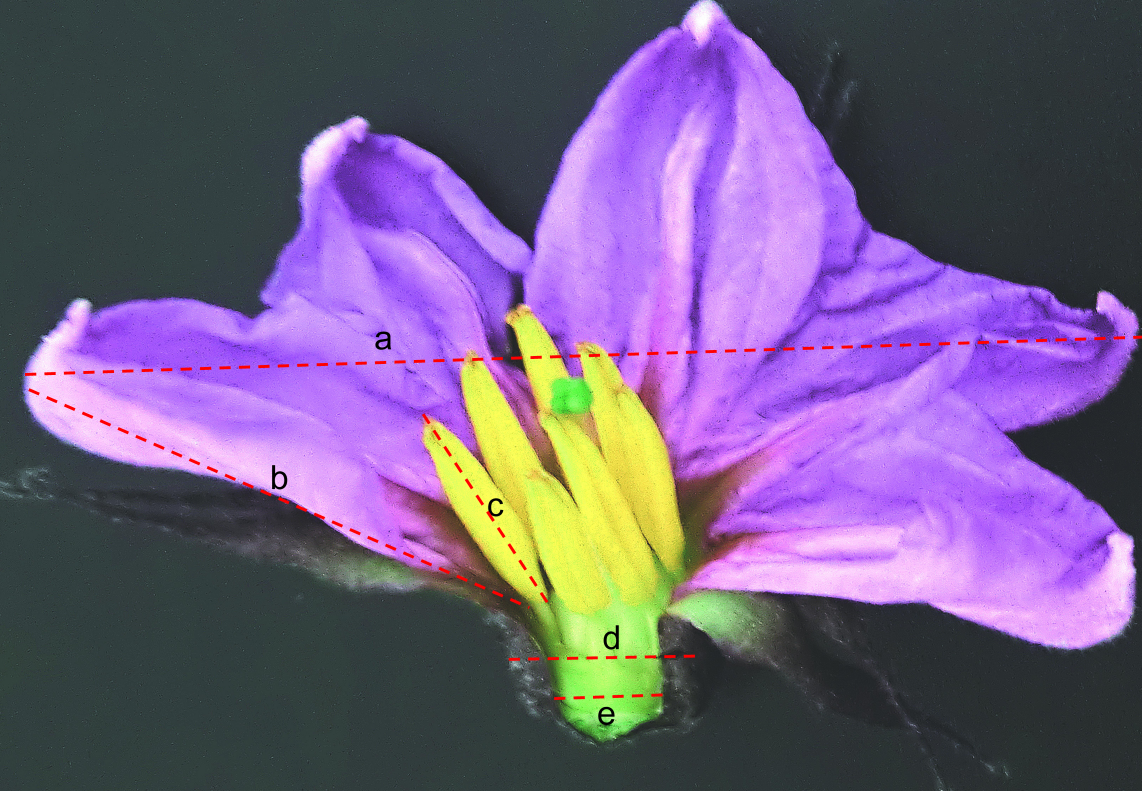


**Fig. S5 The measuring parts in flower morphology.** a Opening degree; b. Petal length; c. Anther length; d. Bud transverse diameter; and e. Ovary transverse diameter.
